# Branched-chain amino acids and risk of stroke: A Mendelian randomization study

**DOI:** 10.3389/fnins.2023.1143718

**Published:** 2023-02-09

**Authors:** Yang Zhang, Yunxia Duan, Miaowen Jiang, Xiaoduo He, Shuaili Xu, Jiaqi Guo, Ming Li, Chen Zhou, Di Wu, Guiyou Liu, Xunming Ji

**Affiliations:** ^1^Department of Neurology, Xuanwu Hospital, Capital Medical University, Beijing, China; ^2^China-America Institute of Neurology, Xuanwu Hospital, Capital Medical University, Beijing, China; ^3^Beijing Institute for Brain Disorders, Capital Medical University, Beijing, China; ^4^Department of Neurosurgery, Xuanwu Hospital, Capital Medical University, Beijing, China

**Keywords:** branched-chain amino acids, stroke, cardioembolic stroke, Mendelian randomization study, single nucleotide polymorphisms

## Abstract

**Background:**

The causality between plasma branched-chain amino acids (BCAAs) levels and stroke remains uncertain and the stratified research on the association between BCAAs levels and subtypes of stroke is not well studied. Therefore, the association of genetically proxied circulating BCAA levels with the risks of stroke and its subtypes was explored by Mendelian randomization (MR) in this study.

**Methods:**

Summary-level data derived from the published genome-wide association studies (GWAS) were employed for analyses. Data for plasma BCAA levels (*n* = 16,596) were obtained from a meta-analysis of GWAS. The MEGASTROKE consortium provided data for ischemic stroke (*n* = 440,328) and its subtypes and data for hemorrhagic stroke were available from 2 meta-analyses of GWAS of European-ancestry groups (intracerebral hemorrhage, *n* = 3,026; subarachnoid hemorrhage, *n* = 77,074). The inverse variance weighted (IVW) method was selected as the primary MR analysis. Supplementary analysis used included the weighted median, MR-Egger regression, Cochran’s Q statistic, MR Pleiotropy Residual Sum and Outlier global test, and leave-one-out analysis method.

**Results:**

According to IVW analysis, 1-SD increment in genetically determined circulating isoleucine was associated with increased risks of cardioembolic stroke (CES) (OR: 1.56, 95% CI: 1.21–2.20, *P* = 0.0007), but not with risks of other stroke subtypes. We could not discover any proof that leucine and valine levels could increase risk of any stroke subtype. All heterogeneity tests produced stable findings, and there was no concrete evidence to indicate the perturbation of horizontal multiplicity.

**Conclusion:**

Increasing plasma isoleucine level had a causal effect on the risk of CES but not on the risk of other stroke subtypes. Further research is needed to identify the mechanisms of the causal associations between BCAAs and stroke subtypes.

## Introduction

Stroke accounts for almost 5% of all disability-adjusted life years and 10% of all deaths worldwide (Feigin et al., 2018). Hypertension, diabetes, and smoking are some of the most commonly described modifiable risk factors for stroke (Boehme et al., 2017). However, stroke is an extremely complex disease with numerous underlying causes. Therefore, an in-depth understanding of the underlying pathophysiology is necessary to develop and optimize preventative methods, as well as to gain insight into the primary risk factors for the various etiological stroke subtypes.

Branched-chain amino acids (BCAAs), which are essential amino acids produced from food including isoleucine, leucine, and valine, are crucial metabolic indicators and are required for optimal growth and function at the cellular and organismal levels (Huang et al., 2011). On the other hand, BCAA metabolic disorders may cause adverse effects on human health, especially on cardiovascular disease (CVD) (Lynch and Adams, 2014; McGarrah and White, 2022). Among various CVD, the metabolism effect of these BCAAs on stroke remains unclear. One observational study found lower BCAA concentration was associated with the severity of cardioembolic stroke and worse neurological outcome (Kimberly et al., 2013). However, another case-cohort study observed that after adjustment for potential confounders, baseline leucine and isoleucine but not valine concentrations were associated with stroke (including ischemic and hemorrhagic stroke) (Ruiz-Canela et al., 2016). The connection between plasma BCAA levels and stroke has not been thoroughly explored due to potential biases such as confounders or reverse causality; hence, it is unknown if BCAAs have a causal influence on the risk of stroke.

**FIGURE 1:**
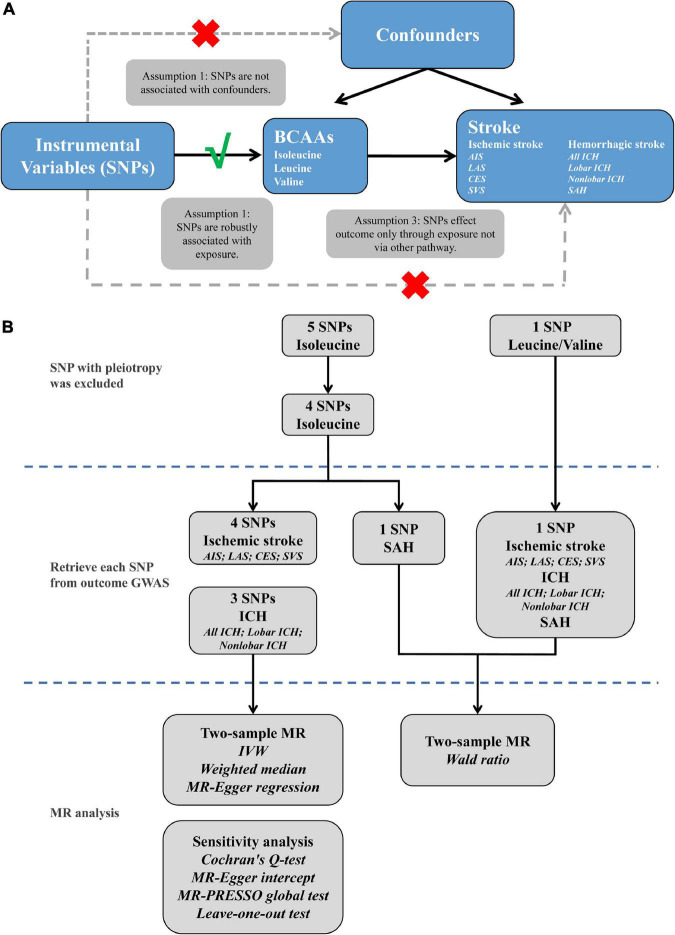
The explanation **(A)** and study flame chart **(B)** of Mendelian randomization analysis for BCAAs and risk of stroke. SNPs, single nucleotide polymorphisms; BCAAs, branched-chain amino acids; AIS, any ischemic stroke; LAS, large artery stroke; CES, cardioembolic stroke; SVS, small vessel stroke; ICH, intracerebral hemorrhage; SAH, subarachnoid hemorrhage; GWAS, genome-wide association studies; MR, Mendelian randomization; IVW, inverse variance weighted; MR-PRESSO, MR pleiotropy residual sum and outlier.

Mendelian randomization (MR) based study that selects genetic variants as instrumental variables to elucidate causality of the disease-associated risk factor could overcome the aforementioned biases. If stroke is causally influenced by BCAAs, then variants affecting blood plasma BCAA levels should influence stroke to some extent. MR analysis is based on three primary assumptions (Emdin et al., 2017): Initially, instrumental variables were significantly associated with exposure; second, no association between instrumental variables and confounders was observed; and finally, the influence of instrumental variables on outcome was entirely through exposure (Figure 1A). A series of two-sample MR analyses were conducted in the present study, based on the summary data from genome-wide association studies (GWAS) of plasma BCAA levels and stroke and its subtypes, to demonstrate the causal influence of BCAAs on the risk of different subtypes of stroke.

## Materials and methods

### Study design

The study frame diagram is shown in Figure 1B. This study was based on publicly available GWAS data about plasma BCAA levels as well as stroke, with more details available in Table 1. In the original trials, proper ethical approval and patient informed agreement could be acquired, and only summary data were utilized in this article.

**TABLE 1:** Summary of detailed information on genome-wide association studies and datasets included in present study.

Exposures /Outcomes	Cohorts or datasets	Participants (European-ancestry)		PMID	Source
BCAAs	Fenland, KORA, TwinsUK	16,596 individuals		27898682	https://ndownloader.figstatic.com/files/6962132
**Ischemic stroke**					
AIS	CHARGE, METASTROKE, SIGN, DECODE, EPIC-CVD, Young Lacunar DNA; SIFAP; INTERSTROKE EUR; HVH1; Glasgow; CADISP; Barcelona; FINLAND; SAHLSIS; MDC; HVH2	Cases	Controls		
		34,217	406,111	29531354	http://www.megastroke.org/download.html
LAS		4,373			
CES		7,193			
SVS		5,386			
**Hemorrhagic stroke**					
ICH					
All ICH	GOCHA; GERFHS I, II; ISGC European Centers	1,545	1,481	24656865	https://cd.hugeamp.org/downloads.html
Lobar ICH					
Non-lobar ICH					
SAH	@neurIST; ARIC; Busselton; Utrecht 1; Utrecht 2; Netherlands (EGA); Doetinchem Cohort Study; Project MinE; French Canadian; Finland (EGA); Finland; NFBC1966; ICAN; PREGO; GAIN; non-GAIN; FIA; NBS; UK Biobank; GOSH controls; GOSH cases; NBS + 1958BBC; HUNT Study	5,140	71,934	33199917	https://doi.org/10.6084/m9.figshare.11303372

The sources of datasets included in present study could be found online. BCAAs, branched-chain amino acids; AIS, any ischemic stroke; LAS, large artery stroke; CES, cardioembolic stroke; SVS, small vessel stroke; ICH, intracerebral hemorrhage; SAH, subarachnoid hemorrhage.

### Data source

The genetic variants associated with plasma BCAA levels were obtained for exposure data from a meta-analysis of GWAS of 16,596 European ancestors (Lotta et al., 2016). Different stroke data were obtained from three publicly available summarized data. The MEGASTROKE consortium used 440,328 individuals of European ancestry to conduct a massive GWAS meta-analysis to identify the genetic variants associated with any ischemic stroke (AIS) (34,217 cases and 406,111 controls). According to the Trial of Org 10172 in Acute Stroke Treatment criteria (Adams et al., 1993), these cases were subtyped as large-artery stroke (LAS, *n* = 4,373), cardioembolic stroke (CES, *n* = 7,193), and small vessel stroke (SVS, *n* = 5,386) (Malik et al., 2018). Summary statistics data for hemorrhagic stroke were available from 2 meta-analyses of GWAS of European-ancestry groups: (1) for intracerebral hemorrhage (ICH), data composed of 1,545 cases (664 lobar and 881 non-lobar) and 1,481 controls (Woo et al., 2014); (2) due to non-traumatic subarachnoid hemorrhage (SAH) was mainly caused by rupture of aneurysms, data of SAH was obtained from a recent GWAS of intracranial aneurysm including 5,140 SAH cases and 71,934 controls (Bakker et al., 2020).

### Selection of instrumental variables

Five distinct genomic regions with single-nucleotide polymorphisms (SNPs) associated with plasma BCAA levels at genome-wide significance level (*P* < 10^–8^) were found in the meta-analysis of GWAS of exposure data. The leading SNPs were selected from each of these genomic regions. The level of isoleucine was associated with five independent SNPs (linkage disequilibrium *r*^2^ < 0.001, and 1 MB from the index variant). Leucine and valine levels were only significantly associated with one SNP (rs1440581). We removed the leading SNP (rs1260326) associated with isoleucine level at the known pleiotropic through PhenoScanner V2 (Supplementary Table 1; Kamat et al., 2019), which is a publicly available GWAS database, because Mendelian randomization assumes no pleiotropic effect beyond that on the risk factor of interest (i.e., BCAAs levels). The strength of genetic variants was then measured using *F-statistics* according to formula of *F* = (N-K-1) × R^2^/K × (1-R^2^), where R^2^ was the proportion of variation in exposure explained by the SNPs, N was the sample size, and K was the number of SNPs in genetically proxied exposure; and *F-statistics* of all remaining SNPs were greater than 10 (Table 2; Burgess and Thompson, 2011). Next, SNPs were extracted from outcome data and all SNPs were not associated with stroke and its subtypes (Table 2). Finally, to conduct MR analyses for BCAAs and stroke, 4 SNPs for isoleucine level and ischemic stroke (AIS, LAS, CES and SVS), 3 SNPs for isoleucine and ICH (all, lobar and non-lobar ICH), 1 SNP for isoleucine and SAH and 1 SNP for leucine/valine levels and ischemic stroke, ICH and SAH were selected (Table 2).

**TABLE 2:** Characteristics of the SNPs associated with BCAAs and stroke.

BCAAs	SNP	Effect allele/Other allele	Effect allele frequency	R^2^	*F-statistics*	Parameter	BCAA	Ischemic stroke				Hemorrhagic stroke			
								AIS	LAS	CES	SVS	ICH			SAH
												All ICH	Lobar ICH	Non-lobar ICH	
Isoleucine	rs7678928	T/C	46%	0.004	67.045	*P*-value	5.6E-19	0.732	0.653	0.069	0.532	0.232	0.169	0.593	0.088
						Beta	0.090	0.003	0.011	0.035	0.014	−0.062	−0.094	−0.033	−0.042
						Standard error	0.013	0.010	0.025	0.019	0.023	0.052	0.068	0.062	0.0244
Isoleucine	rs75950518	C/T	89%	0.002	37.283	*P*-value	2.1E-08	0.457	0.880	0.266	0.313	0.185	0.072	0.599	
						Beta	0.107	0.012	0.006	0.033	−0.036	0.102	0.181	0.047	
						Standard error	0.019	0.016	0.038	0.030	0.036	0.077	0.101	0.090	
Isoleucine	rs1420601	C/T	40%	0.002	39.876	*P*-value	3.7E-08	0.191	0.606	0.133	0.131	0.947	0.177	0.393	
						Beta	0.069	0.015	0.014	0.031	0.038	−0.004	0.095	−0.055	
						Standard error	0.013	0.011	0.027	0.021	0.025	0.053	0.070	0.064	
Isoleucine	rs58101275	G/A	79%	0.002	38.009	*P*-value	2.8E-08	0.025	0.362	0.016	0.850				
						Beta	0.085	0.029	0.028	0.059	0.005				
						Standard error	0.015	0.013	0.031	0.024	0.029				
Leucine	rs1440581	C/T	53%	0.003	54.419	*P*-value	3.9E-25	0.254	0.543	0.028	0.503	0.785	0.266	0.505	0.104
						Beta	0.081	0.012	0.015	0.040	0.016	−0.014	−0.075	0.041	−0.042
						Standard error	0.013	0.010	0.025	0.018	0.023	0.051	0.067	0.061	0.026
Valine	rs1440581	C/T	53%	0.005	79.779	*P*-value	4.4E-24	0.254	0.543	0.028	0.503	0.785	0.266	0.505	0.104
						Beta	0.098	0.012	0.015	0.040	0.016	−0.014	−0.075	0.041	−0.042
						Standard error	0.013	0.010	0.025	0.018	0.023	0.051	0.067	0.061	0.026

SNPs, single nucleotide polymorphisms; BCAAs, branched-chain amino acids; AIS, any ischemic stroke; LAS, large artery stroke; CES, cardioembolic stroke; SVS, small vessel stroke; ICH, intracerebral hemorrhage; SAH, subarachnoid hemorrhage.

### Statistical analyses

No mismatched and palindromic SNPs were found following harmonization of the effect alleles across the GWAS of plasma BCAA levels and stroke, then to calculate MR estimations of BCAAs for stroke and its subtypes, multiple MR methods were applied. As the method with the highest statistical power among all MR techniques, inverse-variance weighting (IVW) was selected as the primary method to provide MR estimation by combining the Wald ratio of each SNP (Burgess et al., 2017). The Wald ratio estimate was used in place of the IVW technique when just one SNP was acting as an instrumental variable. To further analyze the causal association, weighted median and MR Egger regression were then carried out (Bowden et al., 2015, 2016).

Furthermore, sensitivity analysis was conducted to test whether the results were robust and whether the conclusions were reliable. The statistical heterogeneity among SNPs was measured using Cochran’s *Q* test in the IVW technique (*P* < 0.05) (Greco et al., 2015). MR-Egger regression’s intercept was calculated to test for horizontal pleiotropy. The MR-egger regression’s non-zero intercept indicated horizontal pleiotropy (*P* < 0.05) (Bowden et al., 2015). Additionally, the MR-pleiotropy residual sum and outlier (MR-PRESSO) global test was used to identify horizontal pleiotropic outliers (*P* < 0.05) (Verbanck et al., 2018). In addition, using leave-one-out analysis, we assessed the probability that the overall MR estimate was influenced by a single SNP.

Calculated as 0.05/[2 (isoleucine, leucine/valine) × 8 (AIS, LAS, CES, SVS, all ICH, lobar ICH, non-lobar ICH, SAH)] = 3.125 × 10^–3^ for the Bonferroni corrected *P*-value threshold. *P*-values below 3.125 × 10^–3^ were treated as statistically significant. The R software (version 4.2.1) and its associated R package, TwoSampleMR (version 0.5.6), were used to conduct all of the aforementioned statistical studies.

## Results

**FIGURE 2:**
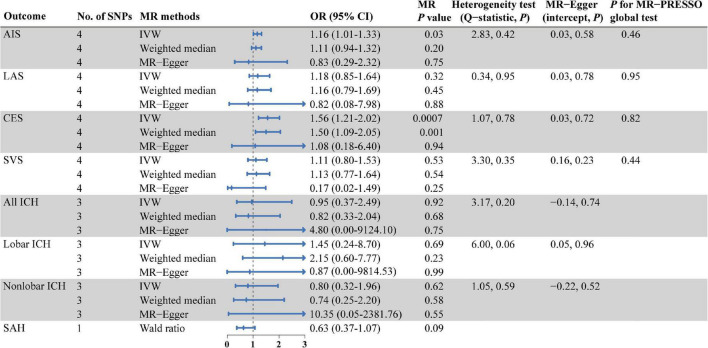
MR estimates from each method of assessing the causal effects of isoleucine level on the risk of stroke and its subtypes. AIS, any ischemic stroke; LAS, large artery stroke; CES, cardioembolic stroke; SVS, small vessel stroke; ICH, intracerebral hemorrhage; SAH, subarachnoid hemorrhage; SNPs, single nucleotide polymorphisms; MR, Mendelian randomization; IVW, inverse variance weighted; OR, odd ratio; CI, confidence interval; MR-PRESSO, MR pleiotropy residual sum and outlier.

**FIGURE 3:**
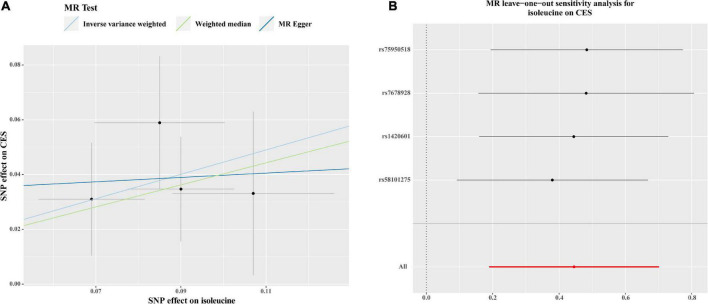
Scatter plots **(A)** and MR leave-one-out sensitivity analysis **(B)** of genetic association with circulating levels of isoleucine against the genetic association with CES risk. **(A)** Each black dot indicates an SNP, plotted by the estimate of SNP on the isoleucine levels and the estimate of SNP on CES risk with standard error bars. The slope of the line represents the causal relationship, and each method has a different line. **(B)** Circles indicate MR estimates for isoleucine on CES using the IVW method if each SNP was omitted in turn. The bars indicate the confident interval. SNP, single nucleotide polymorphism; CES, cardioembolic stroke; MR, Mendelian randomization; IVW; inverse variance weighted.

As shown in Figure 2, there were significant associations detected by IVW between the genetically predisposed difference of 1-SD in isoleucine level and risk of CES (OR: 1.56, 95% CI: 1.21–2.20, *P* = 0.0007), and the data calculated by the weighted median method supported IVW. Although MR Egger regression could not identify the significant association (Figure 2), the estimate was directionally consistent with IVW analysis (Figure 3A). However, no evidence of associations for isoleucine level with risk of AIS (OR: 1.16, 95% CI: 1.01–1.33, *P* = 0.03), LAS (OR: 1.18, 95% CI: 0.85–1.64, *P* = 0.32), SVS (OR: 1.11, 95% CI: 0.80–1.53, *P* = 0.53), all ICH (OR: 0.95, 95% CI: 0.37–2.49, *P* = 0.92), lobar ICH (OR: 1.45, 95% CI: 0.24–8.70, *P* = 0.69) and non-lobar ICH (OR: 0.80, 95% CI: 0.32–1.96, *P* = 0.62) was discovered. Evidence from the weighted median and MR-Egger supported the findings of the IVW (Figure 2). As for SAH, the Wald ratio method detected no genetical effect of isoleucine level (OR: 0.63, 95% CI: 0.37–1.07, *P* = 0.09) (Figure 2). Additionally, Cochran’s *Q* test in IVW and the intercept assessment by MR Egger regression did not reveal any evidence of underlying heterogeneity and pleiotropy, and the MR-PRESSO global test did not identify any outlier SNPs (Figure 2). No one SNP dominated the IVW point estimate, according to the later leave-one-out study (Figure 3B and Supplementary Figure 1). Besides, there was no significant causal effect on risk of stroke from leucine level or valine level detected in Wald ratio analysis (Supplementary Figure 2).

## Discussion

This is the first two-sample MR study that we are aware of that attempts to explore the causal relationship between plasma BCAA levels and the risk of stroke and its subtypes. We investigated the association between plasma levels of isoleucine, leucine, and valine and the risk of AIS, LAS, CES, SVS, all ICH, lobar ICH, non-lobar ICH, and SAH. This MR research revealed that, in contrast to other stroke subtypes, risk of CES was positively associated with a genetic propensity to greater plasma isoleucine levels. Based on a single genetic variant, genetically predicted increased amounts of leucine and valine were not connected to the risk of any subtype of stroke. The results of present study indicate that if the plasma isoleucine level in patients is found to be higher than normal range in clinical examination, the risk of patients getting CES is increased; clinicians can advise patients to reduce the intake of BCAAs, especially isoleucine, and adopt other preventive measures for CES, which optimize preventative methods.

In the pioneering investigations conducted by Cahill et al. over 50 years ago, it was shown that people with obesity and insulin resistance had elevated circulating levels of the BCAAs (Felig et al., 1969). As metabonomics has been developed and applied for decades, increased levels of BCAAs and associated metabolites are regarded as a metabolic indicator of type 2 diabetes, insulin resistance, and obesity in humans (White and Newgard, 2019). The pathophysiology of CVD is closely related to metabolic abnormalities, and independent connections have been found to indicate a direct involvement of BCAAs in CVD (Lynch and Adams, 2014; McGarrah and White, 2022). However, stroke, as a cardiovascular disease with high mortality and disability rate, has not been clearly investigated to be causally associated with BCAAs. Incident stroke was one CVD outcome that was most substantially connected with higher baseline BCAA concentrations in the primary preventive PREDIMED research (Ruiz-Canela et al., 2016). Meanwhile, BCAAs are discovered to be novel markers of CVD development, including stroke, in matched case-control research generated from the population-based Malmö Diet and Cancer Cardiovascular Cohort (MDC-CC) (Magnusson et al., 2013). Besides, total BCAAs only had a marginally significant correlation with stroke in a prospective cohort of American women (Tobias et al., 2018). In contrast, in another study, patients with acute ischemic stroke had lower plasma BCAA levels compared to controls, and a lower BCAA concentration was associated with poorer neurological outcomes (Kimberly et al., 2013). Conclusions from these observational studies were confused and debatable, which may be caused by small sample sizes and potential confounding. Furthermore, it was unable to identify whether the correlation between circulating BCAA levels and stroke risk found in observational studies was a causative one or even a reverse causality. Moreover, most studies limited the differentiation of subtypes of stroke also due to the small sample size. As a result, this MR analysis was conducted, and the reliable conclusion that the circulating level of isoleucine may raise the risk of CES was reached.

Though our findings indicated the causal effect of plasma isoleucine on CES, future studies are needed to determine the causal mechanism. Some clues came from a recent study which found higher plasma BCAA levels may be associated with cardiac arrhythmias *via* mTOR pathway (Portero et al., 2022). Atrial flutter and fibrillation are common arrhythmias and both of them easily lead to blood stasis in the atrium and thrombosis, and once thrombus fall off and block the cerebral vessels, CES might happen. Thus, plasma BCAA levels may induce CES through atrial flutter and fibrillation. This hypothesis needs further exploration of basic research, and mediation analyses of MR analysis can also be helpful.

Our study has some strengths. The first merit of this study was the two-sample MR method’s ability to generate reliable causal association by reducing confounding variables and preventing reverse causality. Additionally, a correlation between BCAA levels and the risk of stroke and its subtypes was examined using MR analysis, which is helpful to distinguish the different effects of BCAA levels on each subtype. Finally, in order to enhance the statistical power, the analyses for the IVs of BCAA levels and stroke were based on high sample sizes. Nevertheless, the following limitations should be understood in the results of this MR analysis. First, the validity of the MR study depended on the chosen genetic instruments. MR analysis may be biased by potential violations of standard instrumental variables assumptions. However, in the present study, no evidence of violations was observed *via* several sensitivity analyses. Second, because population stratification can have an impact on the results of MR studies, only data from summary statistics for people with European ancestry were used; however, there was a limit to how general the observed causal associations could be to other populations with different genetic backgrounds. Finally, there was just one genetic variant each for the isoleucine effect on SAH and leucine as well as valine, which reduced the statistical power to find an association.

## Conclusion

The current study demonstrated that increasing plasma isoleucine level had a causal effect on the risk of CES but not on the risk of other stroke subtypes; in addition, plasma leucine level or valine level had no causal effect on the risk of any stroke subtype. Further research is needed to determine how BCAAs affect the development of stroke subtypes.

## Data availability statement

The original contributions presented in this study are included in this article/Supplementary material, further inquiries can be directed to the corresponding author.

## Ethics statement

Ethical review and approval was not required for the study on human participants in accordance with the local legislation and institutional requirements. Written informed consent for participation was not required for this study in accordance with the national legislation and the institutional requirements.

## Author contributions

YZ participated in designing the study, collecting data, analyzing the data, and writing the manuscript. YD, MJ, XH, SX, and JG were responsible for data collection and processing. YZ and GL did the statistical analysis. ML, CZ, and DW made critical revisions to the manuscript for important intellectual content. XJ was responsible for study supervision, organization of the project, and accepts full responsibility for the finished work. All authors contributed to the article and approved the submitted version.
