# Supplementary material for: Branched-chain amino acids and risk of stroke: A Mendelian randomization study
Source: Front Neurosci. 2023 Feb 9;17:1143718. doi: 10.3389/fnins.2023.1143718 (PMC9947500; doi:10.3389/fnins.2023.1143718)
Supplement: Supplementary file 1 [file Data_Sheet_1.docx]

Supplementary Material

| **Table S1. Associations of the BCAA-associated SNP with other phenotypes at *P* < 0.05 in published genome-wide association studies** | | | | |
| --- | --- | --- | --- | --- |
| **BCAAs** | **SNP** | **Phenotype** | ***P* value** | **PMID** |
| **Isoleucine** | **rs1260326*** | Triglycerides | 2.0E-239 | 24097068 |
|  |  | Serum urate | 1.3E-44 | 23263486 |
|  |  | C-reactive protein | 3.8E-43 | 23263486 |
|  |  | Fasting glucose | 2.2E-41 | 22885924 |
|  |  | HDL cholesterol | 6.3E-36 | 19936222 |
|  |  | Coagulation factor VII | 1.7E-28 | 21676895 |
|  |  | Total cholesterol | 7.0E-27 | 20686565 |
|  |  | Fasting insulin | 2.7E-22 | 22885924 |
|  |  | Chronic kidney disease | 3.0E-14 | 20383146 |
|  |  | Gout | 2.0E-12 | 25646370 |
|  |  | Serum creatinine | 3.4E-12 | 20383146 |
|  |  | Apolipoprotein C | 8.7E-12 | 19060906 |
|  |  | Height | 1.4E-11 | 25282103 |
|  |  | Age at menopause | 1.0E-9 | 26414677 |
|  |  | Type 2 diabetes | 3.7E-9 | 26551672 |
|  |  | Plasma palmitoleic acid | 3.8E-9 | 23362303 |
|  |  | Alcohol consumption | 2.3E-8 | 28485404 |
|  |  | Coffee consumption | 7.0E-8 | 25288136 |
|  |  | Leptin | 3.6E-7 | 26833098 |
|  |  | HOMA insulin resistance | 9.2E-7 | 20081858 |
|  |  | Apolipoprotein A | 9.9E-7 | 19802338 |
|  |  | Apolipoprotein B | 1.6E-6 | 19802338 |
|  |  | Crohn's disease | 2.3E-6 | 18587394 |
|  |  | Body mass index | 4.6E-5 | 25673413 |
|  |  | LDL cholesterol | 2.3E-4 | 20686565 |
| Isoleucine | rs7678928 | Serum urate | 2.4E-4 | 23263486 |
|  |  | Irritable bowel syndrome | 0.002 | 18587394 |
|  |  | Geographic atrophy | 0.005 | 23455636 |
|  |  | Bipolar disorder | 0.007 | 22182935 |
|  |  | Age-related macular degeneration | 0.013 | 23455636 |
|  |  | Crohn's disease | 0.016 | 23128233 |
|  |  | Obesity class 1 | 0.022 | 23563607 |
|  |  | 2 hour glucose | 0.044 | 20081857 |
| Isoleucine | rs75950518 | Lumber spine bone mineral density | 0.019 | 26367794 |
|  |  | Femoral neck mineral density | 0.021 | 26367794 |
| Isoleucine | rs58101275 | Schizophrenia | 0.004 | 25056061 |
|  |  | Femoral neck mineral density | 0.004 | 26367794 |
|  |  | Crohn's disease | 0.015 | 26192919 |
|  |  | Neuroticism | 0.026 | 27089181 |
| Isoleucine | rs1420601 | Major depression disorder | 0.003 | 26176920 |
|  |  | Schizophrenia | 0.007 | 25056061 |
|  |  | Myocardial infarction | 0.037 | 26343387 |
| Leucine/ Valine | rs1440581 | Metabolite levels | 1.0E-16 | 24816252 |
|  |  | Serum Fischers ratio | 2.0E-16 | 22286219 |
|  |  | Serum alanine/valine and leucine | 3.8E-15 | 22286219 |
| *Only the major phenotypes are listed because of the large number of pleiotropic associations searched from PhenoScanner V2 (http://www.phenoscanner.medschl.cam.ac.uk/) (Kamat et al., 2019). This SNP is excluded from the analysis. BCAAs, branched-chain amino acids; SNP, single nucleotide polymorphism. | | | | |


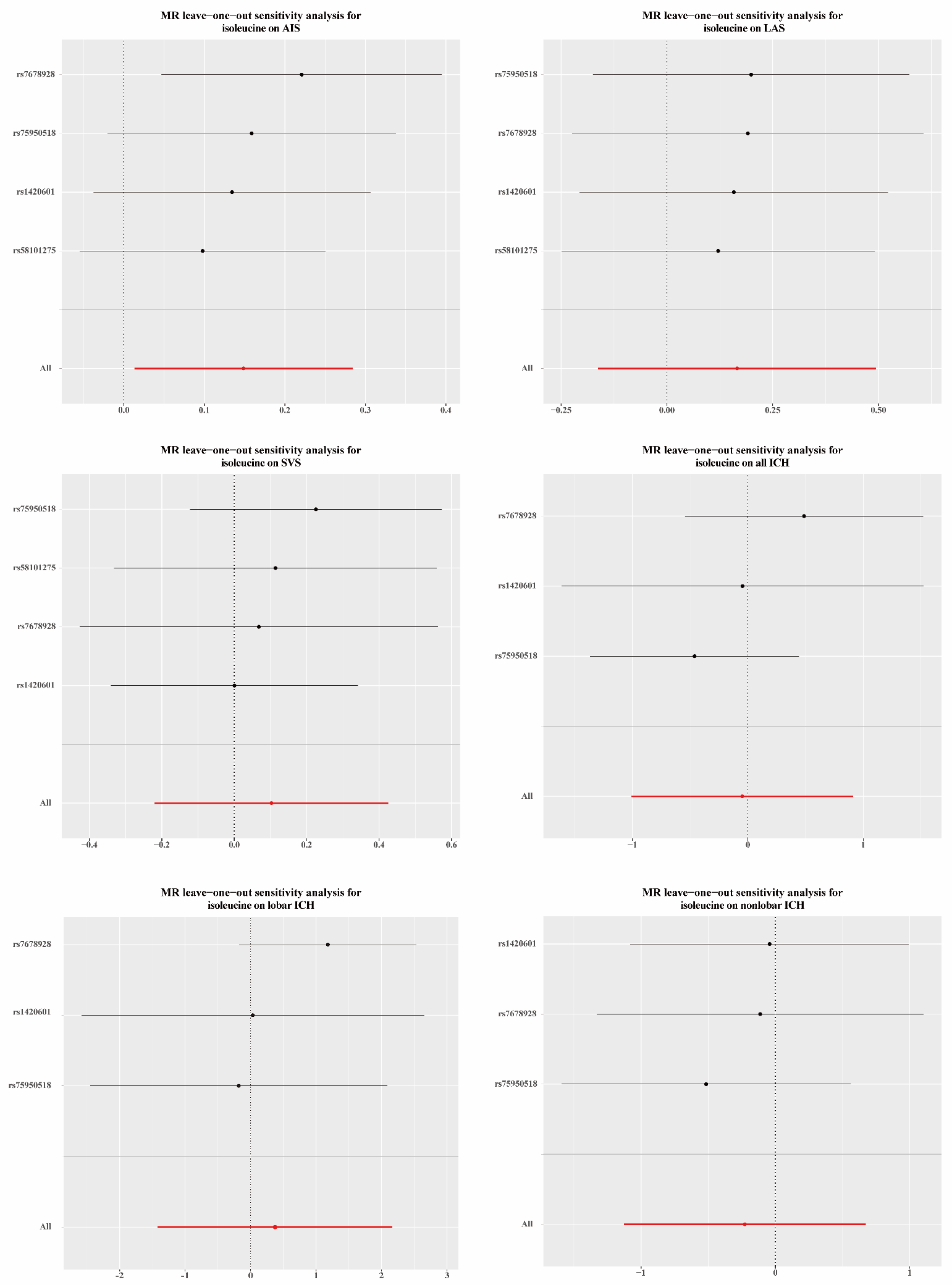
**Figure S1. MR leave-one-out sensitivity analysis for isoleucine on risk of stroke and its subtypes.** Circles indicate MR estimates for isoleucine on risk of stroke and its subtypes using IVW method if each SNP was omitted in turn. The bars indicate the confident interval. MR, Mendelian randomization; AIS; any ischemic stroke; LAS, large artery stroke; SVS, small vessel stroke; ICH; intracerebral hemorrhage; IVW; inverse variance weighted; SNP, single nucleotide polymorphism.


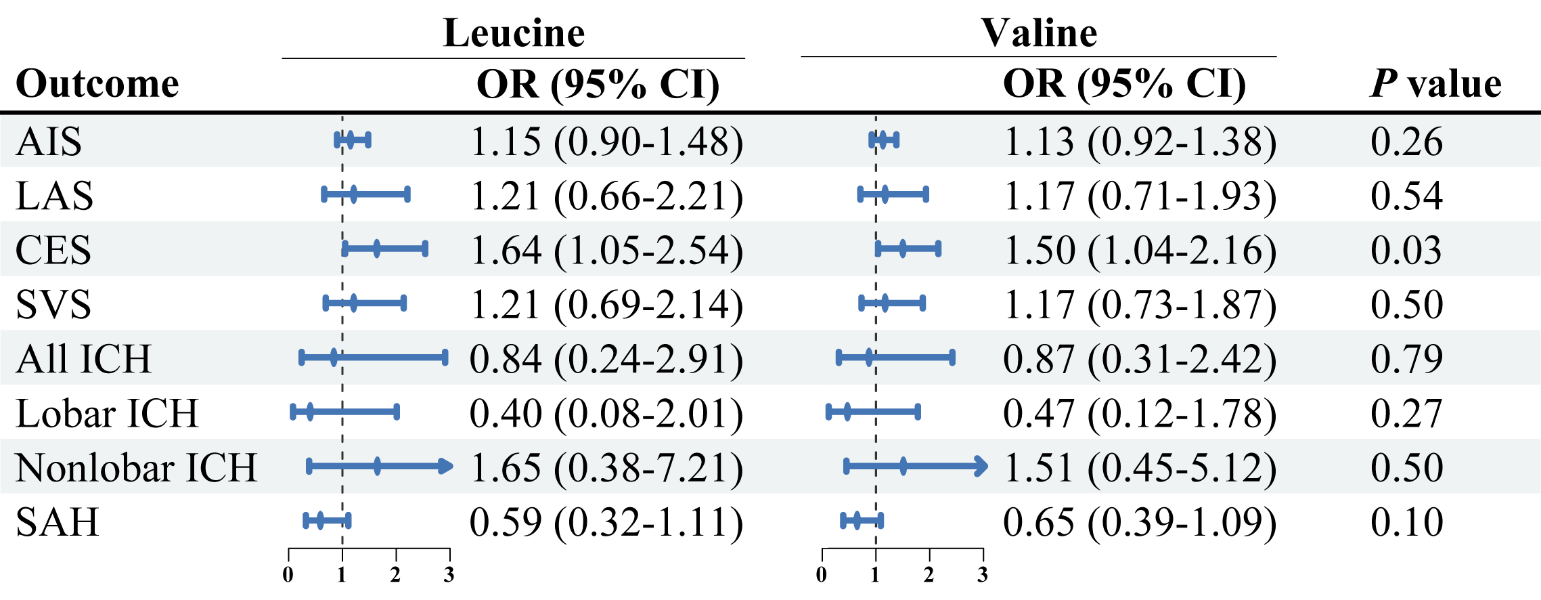


**Figure S2. MR estimates from each method of assessing the causal effects of leucine/valine level on the risk of stroke and it subtypes.** AIS, any ischemic stroke; LAS, large artery stroke; CES, cardioembolic stroke; SVS, small vessel stroke; ICH, intracerebral hemorrhage; SAH, subarachnoid hemorrhage; OR, odd ratio; CI, confidence interval.

**Reference**

Kamat, M. A., Blackshaw, J. A., Young, R., Surendran, P., Burgess, S., Danesh, J., et al. (2019). PhenoScanner V2: an expanded tool for searching human genotype-phenotype associations. Bioinformatics 35, 4851-4853. doi: 10.1093/bioinformatics/btz469.
